# Integrated approach for identifying and evaluating the quality of *Marsdenia tenacissima* in the medicine market

**DOI:** 10.1371/journal.pone.0195240

**Published:** 2018-04-06

**Authors:** Ning Yu, Yu-long Wei, Yue Zhu, Ning Zhu, Yan-li Wang, Hai-ping Zhang, Ai-dong Sun

**Affiliations:** 1 College of Biological Sciences and Technology, Beijing Forestry University, Beijing, China; 2 Beijing Key Laboratory of Forest Food Processing and Safety, Beijing Forestry University, Beijing, China; Chinese Academy of Medical Sciences and Peking Union Medical College, CHINA

## Abstract

The accurate identification and quality evaluation of herbal medical plants is highly necessary to ensure their safety and efficacy. In present study, a new strategy combining DNA barcoding techniques with thin-layer chromatography (TLC) and high-performance liquid chromatography (HPLC) was proposed to facilitate the identification and quality control of *M*. *tenacissima*. In present work, the internal transcribed spacer 2 (ITS2) barcode was successfully used to identify 58 *M*. *tenacissima* samples and its adulterants. TLC successfully identified the other three *M*. *tenacissima* samples that failed to produce ITS2 regions. An adulterant was found in all the 62 samples. Moreover, the content of active medicinal ingredients is important for herbal plants quality. The content of tenacissoside H (TS-H) of *M*. *tenacissima* samples was determined by HPLC to range from 0.39% to 1.09%, which meets the criterion of the Chinese Pharmacopoeia. Thus, DNA barcoding coupled with TLC and HPLC is very promising to identify and evaluate the quality of *M*. *tenacissima* in the medicine market.

## Introduction

Medicinal herbal plant is one of the important agricultural economy crops. The demand for herbal plants has rapidly increased in the last decades. Medicinal plants contribute 80% of raw materials for traditional drug preparation. The worldwide trade for medicinal plants and its products is estimated to be $60 billion annually. However, there has been an increase of unethical commercial trades, whereby the authentic medicinal herb is substituted by less effective and sometimes deleterious herbs and unlabelled fillers [1, 2]. The safe use of herbal medicines requires prior authentication of the raw herbal materials used to make them. Thus, the rapid and accurate identification of herbal medical plants is highly necessary to ensure their safety and efficacy.

DNA barcoding is a process that uses a short DNA sequence from a standard genome to identify existing species and discover unknown ones [3–5]. DNA barcoding is a reliable solution to authenticate raw herbal medicinal plants and establish quality control levels within the market [6, 7]. ITS2, a section of the nuclear ribosomal ITS region, has been highly proposed for species identification because of its universal primers, short length, and taxonomic signatures in evolutionary analysis [8–10]. The ITS2 region has been widely applied in identifying herbal medicinal species [11–13]. Meanwhile, the ITS2 barcode is a convenient and effective tool in herbal market supervision [14, 15].

In addition to molecular methods, physical and chemical methods are also important for the quality control of herbal medicinal plants. Thin-layer chromatography (TLC) is usually applied for the qualitative analysis of target compounds and visual fingerprints [16]. TLC is a simple and fast method for medicinal herbal drug identification and is included in the Chinese Pharmacopoeia. Moreover, active medicinal ingredients is an important index for the quality of raw herbal plants, high-performance liquid chromatography (HPLC) is used to qualitatively and quantitatively detect the constituents of herbal medicinal plants. HPLC is highly suitable for the quality assurance of various herbal products [17–19].

*Marsdenia tenacissima* (Roxb.) Wight et Arn. (Asclepiadaceae), well known as “Tong-guan-teng”, is a traditional Chinese medicine that has attracted considerable attention and has been widely applied in clinical treatment because of its antitussive, expectorant, antiasthma, heat-clearing, and detoxifying effects [20, 21]. In addition, *M*. *tenacissima* exerts significant curative effects on esophageal cancer, gastric cancer, lung cancer, and hepatocellular carcinoma. The *M*. *tenacissima* extract Xiao’aiping has been produced and marketed [22–24]. However, the stems of *M*. *tenacissima* sold in medicine markets are usually dried, sliced, shredded, and processed, thereby complicating the traditional morphological identification of this species because of the absence of identifying characteristics. In addition, *M*. *tenacissima* is always substituted by *Telosma cordata* (Asclepiadaceae) and *Fissistigma polyanthum* (Annonaceae) in folk medicine, which may lead to poor health treatment outcomes and even cause major medical accidents. Therefore, accurate identification and quality evaluation of *M*. *tenacissima* are particularly important to ensure clinical safety.

In this study, DNA barcoding was coupled with TLC and HPLC to identify and evaluate the quality of *M*. *tenacissima* in herbal medicine markets. Our results demonstrate that the integrated method is effective.

## Experiment section

### Plant materials

Sixty-two *M*. *tenacissima* samples were collected from medicine markets and drug stores in Shanxi, Yuzhou, Jiangxi, Baoding, Yunnan, Bozhou, Shanghai, Guizhou, Xingning, Chengdu, and Yulin. Other published sequences of *M*. *tenacissima* and its adulterants were downloaded from GenBank (S1 Table). Voucher specimens of the collected samples were deposited at Beijing Forestry University, China.

### Molecular analyses

The surfaces of the collected samples were scraped and then wiped using 75% ethanol. Total genomic DNA was extracted from the crushed materials with the Plant Genomic DNA Kit (TIANGEN Biotech Co., China). The ITS2 sequences were amplified with the universal primers: ITS2-2F, 5’-ATGCGATACTTGGTGTGAAT-3’ and ITS2-3R, 5’-GACGCTTCTCCAGACTACAAT-3’. PCR amplifications volumes containing 12.5 μL of 2 × EasyTaq PCR SuperMix (Beijing Baierdi Biothch Co., China), 8.5 μL of molecular grade water, 1 μL of each primer (2.5 μM), and 2 μL of the DNA template. PCR condition was 94°C for 5 min, followed by 40 cycles at 94°C for 45 s, 56°C for 45 s, 72°C for 1.5 min, and a final extension step at 72°C for 10 min [10, 25]. The original sequences were trimmed and assembled with CodonCode Aligner 6.0.2 (CodonCode Co., USA). The ITS2 sequences were trimmed with the Hidden Markov Model to remove the 5.8S and 28S regions [26]. All sequences were aligned by MUSCLE [27]. The aligned length, GC content range, variable sites, and genetic distances based on the Kimura 2 parameter (K2P) model were calculated and a neighbor-joining (NJ) tree with 1000 bootstrap text replicates was constructed with MEGA 5.2.2 [28]. DNA barcoding gaps were calculated to compare the distributions of interspecific divergence and intraspecific variation [11, 29]. The ITS2 sequences were transformed into 2D images by using the quick response (QR) code approach [30].

### TLC identification

In a 15 mL centrifuge tube, 1 g of sample powder was dissolved in 10 mL of methanol and then subjected to ultrasonic treatment (100 W, 50 KHz) for 30 min. The extract was filtered, and the filtrate was evaporated to dryness with a rotary evaporator (Yarong Bio., Shanghai, China). The residue was dissolved in 10 mL of water and then added with 10 mL of chloroform. The mixture was shaken for better extraction. The chloroform extract was concentrated to 1 mL, serving as the test solution. The contrast herb (genuine *M*. *tenacissima*) solution was prepared using the above method. A 0.5 mg sample of tenacissoside H (TS-H) was dissolved in 1 mL of chloroform to prepare a 0.5 mg/mL reference solution. The three solutions with 2 μL imbibition were placed on the same silica gel G thin plate (10 × 100 mm) with a 0.5 cm horizontal distance between samples. The developing agent was a mixture of chloroform, acetone, and methanol with a ratio of 20:1:1, sprayed with vanillin/sulfuric acid solution after drying on the G thin plates and then heated at 105°C until spot colors were observed [21].

### HPLC determination

In a 50 mL centrifuge tube, 0.5 g of sample powder was dissolved in 40 mL of methanol, subjected to ultrasonic treatment (100 W, 50 KHz) for 45 min, and then allowed to cool. The loss was compensated with methanol. The extract was filtered, 25 mL of the filtrate was evaporated to dryness, and the residue was dissolved in 2 mL of methanol for detection.

The diagnostic component TS-H was measured using a Shimadzu HPLC with a UV detector. Chromatographic separation was performed on a XDB-C18 column (4.6 × 250 mm, Agilent Eclipse, USA) at a column temperature of 35°C using the mobile phase acetonitrile: water (50:50) with 20 μL of injected mass [21, 31]. Standard solution containing TS-H at a concentration of 1.5 mg/mL and different volumes of standard solution were injected to construct the calibration curve.

## Results

### Amplification, sequencing, and alignment

The PCR amplification success rate of the ITS2 region from the collected samples was 95.16%. All purified PCR products were sequenced, and 100% of bidirectional trace files were high quality. All generated sequences were submitted to GenBank (Additional file 1). The ITS2 sequence lengths of 58 *M*. *tenacissima* samples used in the analyses ranged from 235 bp to 237 bp. Three haplotypes of *M*. *tenacissima* were generated, and C bases were inserted at the 13 sites of the sequence. The GC content varied from 65.10% to 65.40%, the aligned length was 273 bp, and the proportion of variation sites was 45.79% (Table 1).

**Table 1:** Characteristics of *ITS2* sequence of *M*. *tenacissima* and its adulterant.

	*ITS2*
Amplification efficiency (%)	95.16
Sequencing efficiency (%)	100
Length of *M*. *tenacissima* (bp)	235–237
Aligned length (bp)	273
G+C content range of *M*. *tenacissima* (%)	65.10–65.40(65.30)
Number (and %) of variable sites in all taxa	125(45.79%)

### Genetic distances and NJ tree

The genetic distances of the 63 ITS2 sequences were calculated according to the K2P model. The intraspecific distance of *M*. *tenacissima* was 0.000. The distance between *M*. *tenacissima* and *T*. *cordata* was 0.3097, which was the minimum interspecific distance (Table 2).

**Table 2:** Analysis of intra/inter-specific divergence of the ITS2 sequences.

	K2P value
Intra-specific distance of *M*. *tenacissima*	0.0000
Inter-specific distance between *M*. *tenacissima* and *T*.*cordata*	0.3097
Inter-specific distance between *M*. *tenacissima* and *F*.*polyanthum*	0.4192
Inter-specific distance between *M*. *tenacissima* and *T*.*sinensis*	0.6623

**Fig 1:**
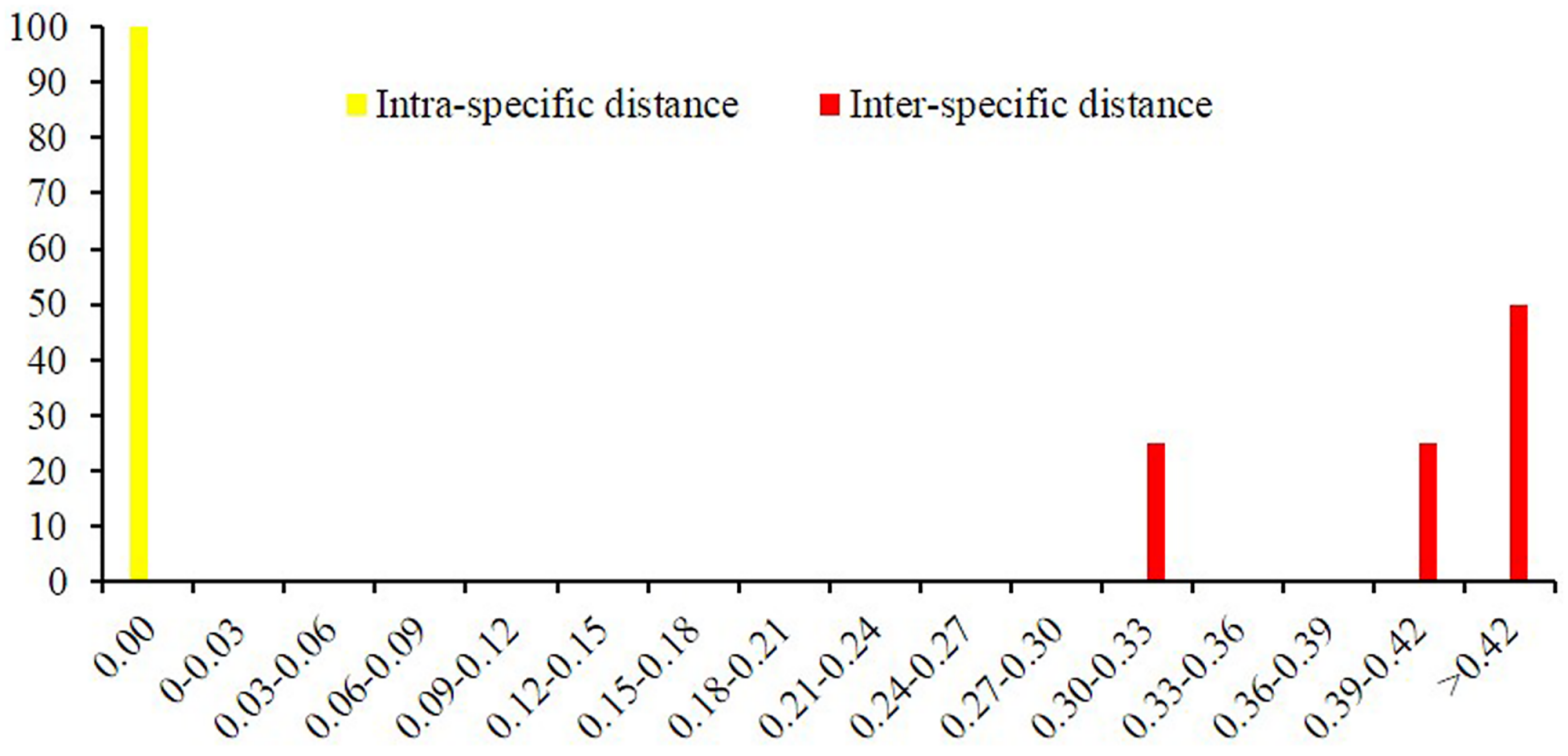
Relative distribution of interspecific divergence and intraspecific variation for ITS2 region. x-axes relate to Kimura 2 parameter distances arranged in intervals, and the y-axes correspond to the percentage of occurrences.

To examine inter- and intraspecific variations, the distribution of genetic distance in *M*. *tenacissima* and its adulterants was investigated in a class of 0.03 units. The minimum interspecific distance was considerably larger than the intraspecific distance; hence, no overlap and clear species boundaries were observed (Fig 1).

**Fig 2:**
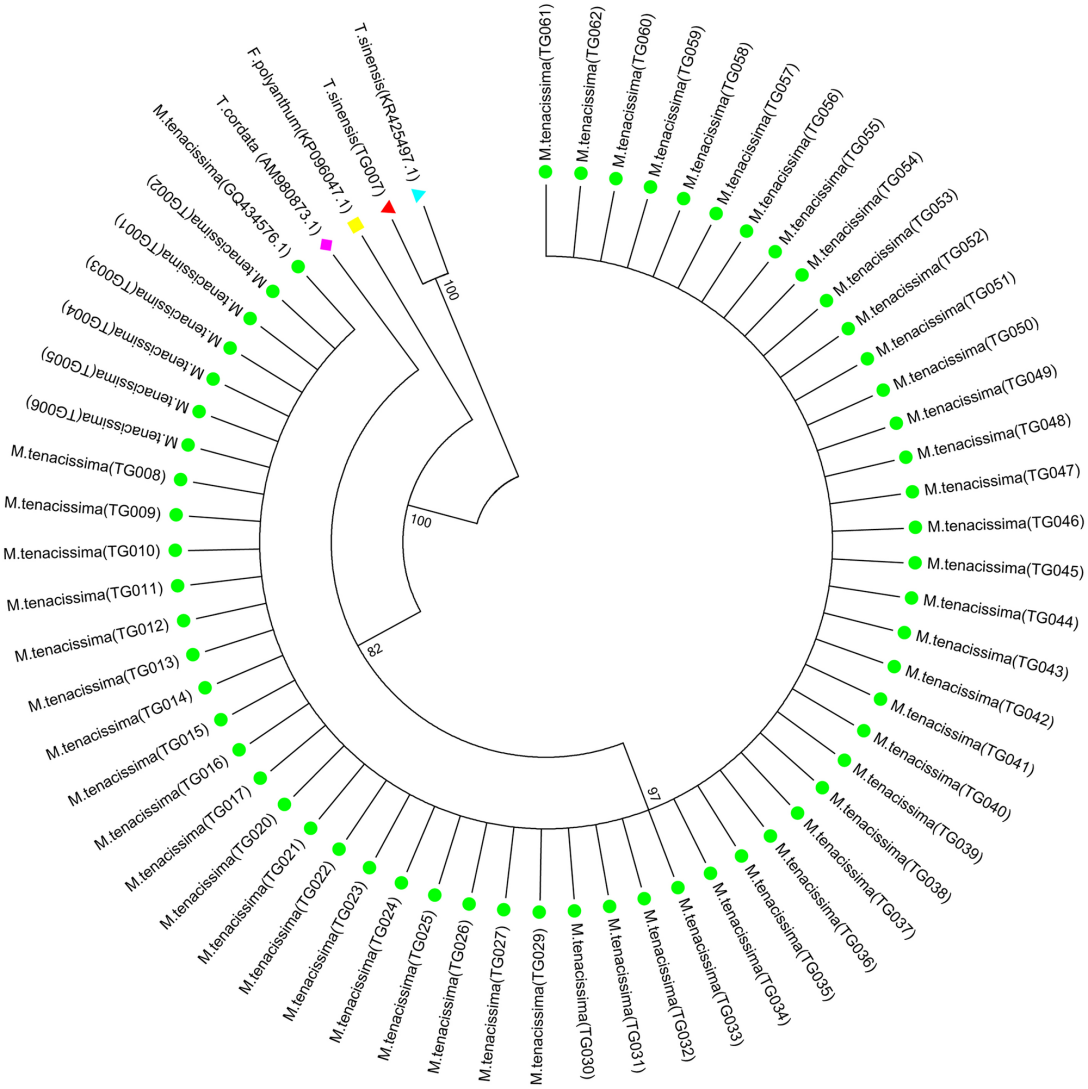
NJ tree based on *ITS2* sequences for *M*. *tenacissima* and its adulterants. Phylogenetic relationships among *M*. *tenacissima* and its adulterants based on the genetic distances. Numbers at nodes indicate the bootstrap values, and the bootstrap was with 1000 replicates for each branch, the values lower than 50% were hidden.

The NJ tree demonstrated that all the *M*. *tenacissima* samples formed one clade, whereas the adulterants clustered into other clades. Therefore, the NJ tree method can clearly distinguish between *M*. *tenacissima* and its adulterants. Notably, TG007, which was confirmed to be *Tinospora sinensis*, clustered into the same clade with the *T*. *sinensis* sequence downloaded from GenBank (Fig 2).

### Two-Dimensional DNA barcoding for *M*. *tenacissima*

**Fig 3:**
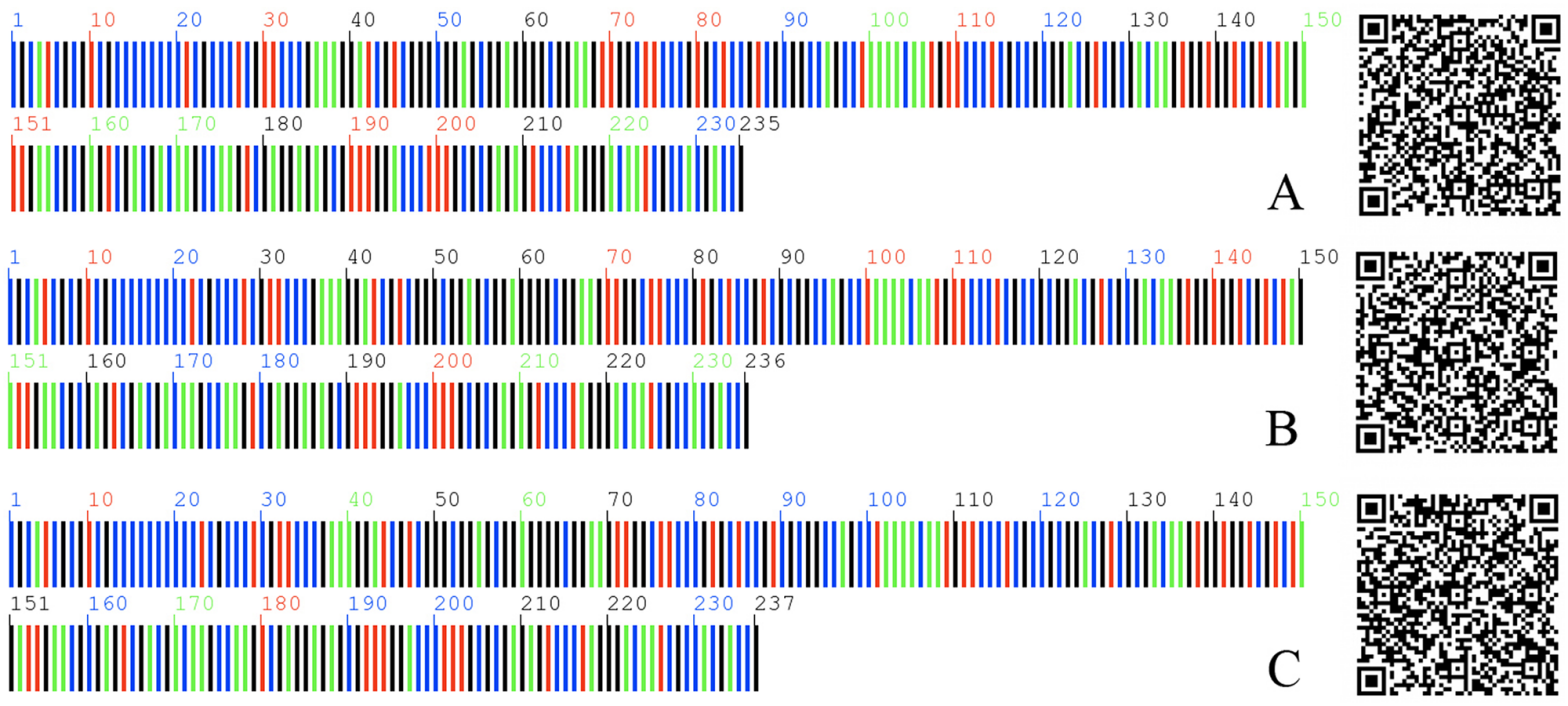
Two dimensional barcode for different haplotypes *M*. *tenacissima*. The ITS2 sequences of *M*. *tenacissima* were translated into two dimensional barcode. Different color represent different bases (green, A; red, T; blue, C; black, G). Numbers represent the sequence length, A 235bp; B 236 bp; C 237 bp. Sequences can be obtained by scanning the right QR code.

With the QR code method, the ITS2 sequences of three haplotypes of *M*. *tenacissima* were transferred into 2D DNA barcoding. In the left of the 2D DNA barcoding, different bases were represented by different colors, and the number represents the sequence length. The sequences of *M*. *tenacissima* were observed by scanning the right image through the scanner (e.g., mobile terminal) (Fig 3). Then, the sequences were uploaded to the Internet for species identification.

### TLC chromatograms

**Fig 4:**
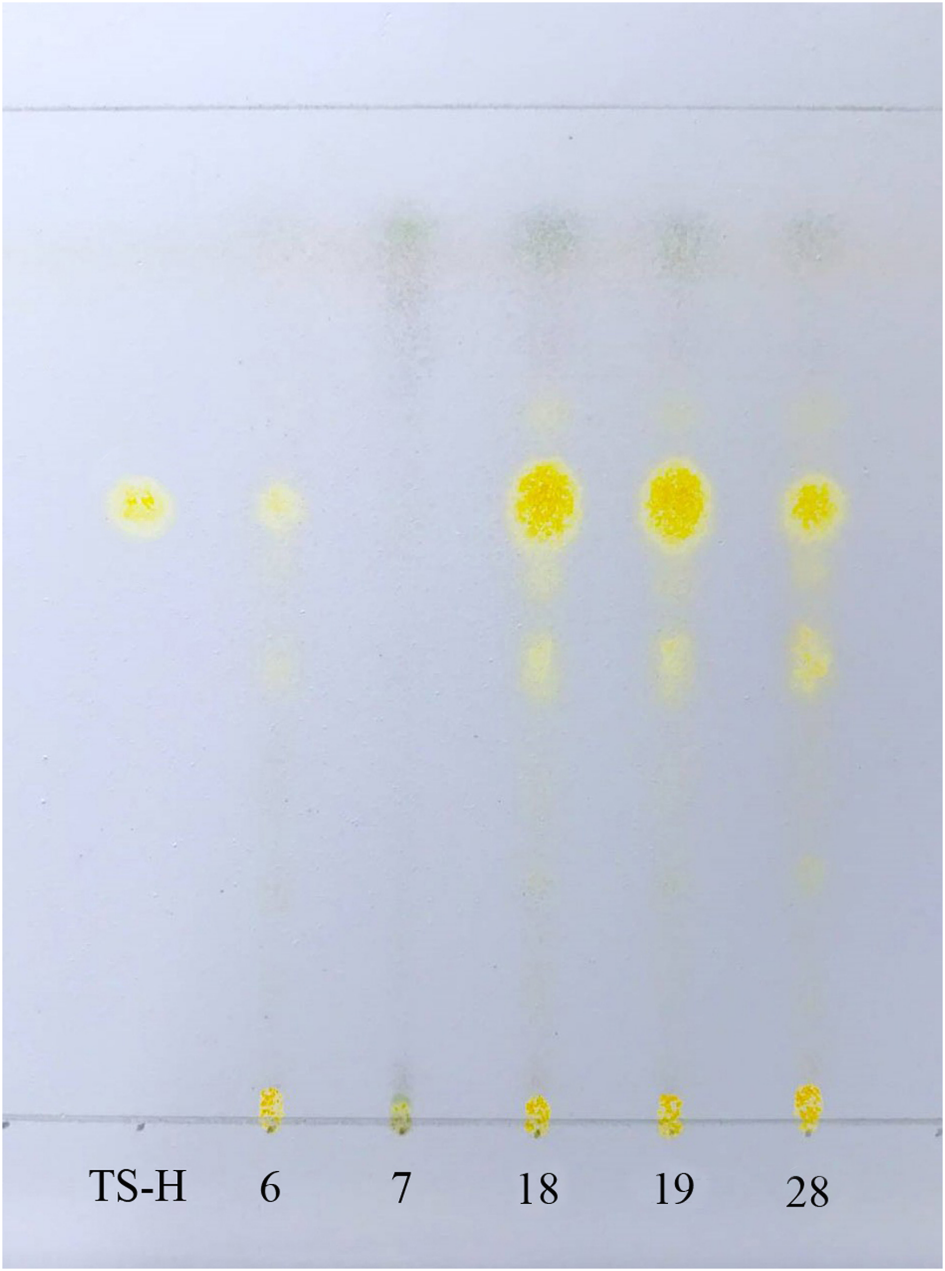
TLC chromatograms of *T*. *sinensis* and three *M*. *tenacissima* failed to produce ITS2 regions. Tenacissoside H is a specific substance of *M*. *tenacissima*, the standard product was used as a control. Identifying species according to whether there is a same color reaction in the same position on thin-layer chromatography.

High-quality ITS2 sequences of TG018, TG019, and TG028 were not obtained. TLC results indicated that these samples exhibited the same yellow spots in the same positions as the control herb material (TG006, random selection) and the standard solution TS-H. The spots were clear and distinguished, and the result was reproducible. For the TG007 sample, no yellow spots were observed at the same position on the G thin plate (Fig 4).

### HPLC analysis

**Fig 5:**
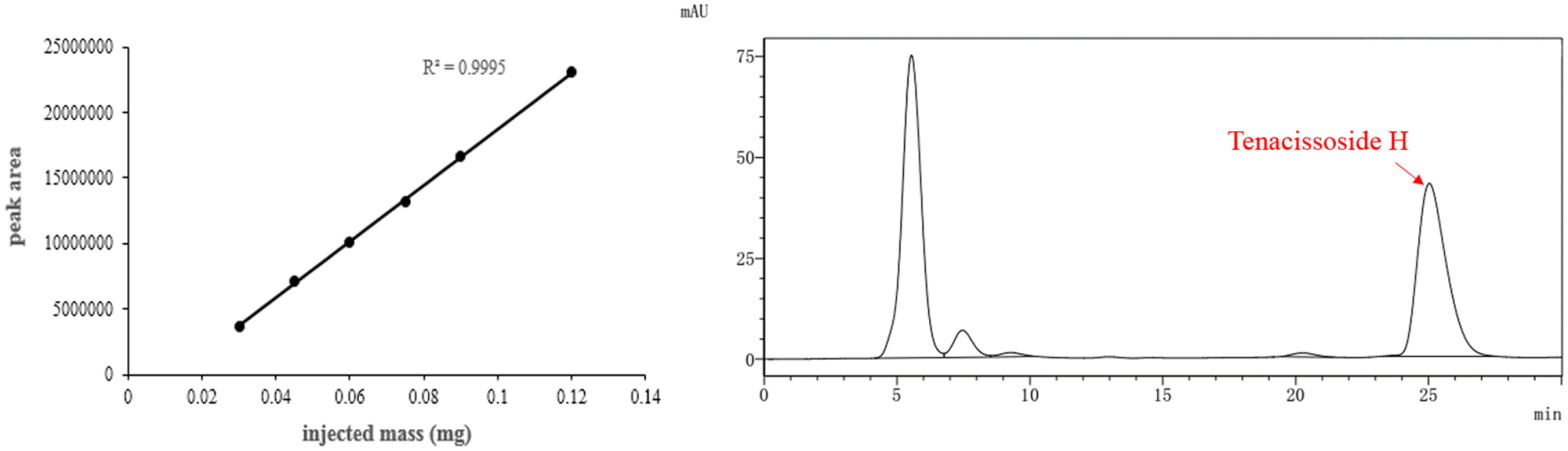
Linearity curves of TS-H between peak area and injected mass. Standard curve of Tenacissoside H was conducted according to peak area and injected mass. Chromatographic peak and appearance time of Tenacissoside H in high-performance liquid chromatography was illustrated.

The working calibration curves presented high linearity ranging from 0.03 mg to 0.12 mg (r = 0.9995) for TS-H, and the regression curve was Y = 2×10^8^X-3×10^6^ (r = 0.9995), where X is the injected mass (mg) and Y is the peak area of the TS-H standard (Fig 5). All 62 stems of the samples were prepared in triplicate with the HPLC method described above, and the results are shown in Table 3. The TS-H contents ranged from 3.872 mg/g to 10.882 mg/g (Table 3).

**Table 3:** The contents of TS-H of the 62 samples (mean±SD, mg/g, n = 3).

NO.	Contents	NO.	Contents	NO.	Contents	NO.	Contents
1	5.569±0.067	17	5.454±0.020	33	7.711±0.037	49	7.065±0.085
2	6.231±0.027	18	9.233±0.113	34	6.750±0.077	50	6.195±0.065
3	5.956±0.047	19	10.719±0.017	35	6.532±0.080	51	6.186±0.082
4	5.671±0.061	20	6.857±0.057	36	6.019±0.044	52	6.937±0.095
5	5.423±0.078	21	5.246±0.003	37	6.167±0.008	53	5.789±0.059
6	3.872±0.032	22	7.060±0.092	38	5.976±0.039	54	6.553±0.084
7	----	23	5.436±0.013	39	5.211±0.026	55	5.854±0.020
8	6.868±0.042	24	9.954±0.088	40	5.460±0.058	56	6.089±0.061
9	4.825±0.015	25	10.281±0.105	41	7.484±0.056	57	5.204±0.055
10	10.880±0.018	26	10.697±0.065	42	10.709±0.048	58	4.585±0.058
11	10.882±0.112	27	9.381±0.095	43	8.415±0.090	59	4.735±0.022
12	6.293±0.073	28	6.920±0.032	44	5.897±0.040	60	4.353±0.058
13	5.812±0.040	29	6.825±0.058	45	5.792±0.065	61	6.196±0.070
14	5.765±0.033	30	6.756±0.051	46	5.909±0.039	62	4.530±0.020
15	6.070±0.035	31	5.996±0.072	47	7.607±0.068	**--**	----
16	6.019±0.043	32	5.684±0.091	48	5.403±0.060	**--**	----

## Discussion

### DNA barcoding for identification and supervision practice

Herbal products sold in the marketplace may suffer from contamination and may be acquired from unethical practices [32]. The adulteration and substitution of herbal drugs may lead to a decline in consumer faith [33]. New et al. suggested that DNA barcoding should be embraced in the herbal industry for identifying herbal products via investigating raw materials [32]. In the present study, the ITS2 barcode could successfully distinguish *M*. *tenacissima* from its adulterants, meanwhile, we found one adulterant of *M*. *tenacissima* after using the ITS2 barcode. This adulterant was identified to be *T*. *sinensis* and not the usual substitutes *T*. *cordata* and *F*. *polyanthum*. This result precisely verified the practical application of DNA barcoding for the supervision in medicine markets.

At present, “DNA barcode” only refers to DNA sequences, which demonstrate some limitations in practice, including storage, recognition, and retrieval of DNA barcode information. Liu et al. suggested the use of the QR code for DNA barcoding sequences. Adopting the QR code as the representation of the DNA barcode promotes the practical application of DNA barcoding [30]. The ITS2 sequences of three haplotypes of *M*. *tenacissima* were translated into 2D DNA barcodes, which rendered the identification rapid and convenient in practice.

### DNA barcoding together with TLC and HPLC for quality control

DNA barcoding is a powerful tool for alleviating the global concern about the clinical safety of herbal medicines [7]. However, the genomic DNA of raw herbal materials may seriously degrade and become difficult to amplify because of harvesting, processing mode, environmental factors, and storage duration. Lo et al found that DNA could still be amplified for DNA identification with extensive processing and boiling [34]. In addition, species-specific primers designed for selected regions would be a better solution to differentiate between genuine and closely related material [35]. However, for some species, the serious degradation of DNA leads to no DNA amplification is also a reality. In addition, some stubborn secondary metabolisms may also affect the amplification. In this scenario, DNA barcoding cannot identify the species. TLC is a simple, rapid, and cheap physicochemical method to investigate the components of a mixture and identify its compounds. In addition, TLC has been applied to confirm the purity and identify the isolated compounds [36]. Certainly, TLC has its limitations. For instance, adulterants contain the same effective ingredient with authentic herbal products, in such a situation, they cannot be accurately identified by TLC merely relying on a specific component spot. For *M*. *tenacissima*, the TS-H is a unique ingredient. In this study, TG018, TG019, and TG028 failed to produce ITS2 regions, the A260/A280 values of the three samples were 1.29, 2.62, 2.53, respectively. We attribute the failure to low DNA extraction and DNA degradation. We tried to remove sticky residues by washing the precipitants with wash buffer three times, however, some residues could not be removed, which make it difficult to extract DNA. These three samples and one adulterant were successfully identified by TLC. All the collected samples were effectively authenticated through DNA barcoding and TLC. TLC chromatograms were used to complement DNA barcoding in identifying *M*. *tenacissima*.

The active ingredient content is a significant index in evaluating the quality of medicine herbs and plant metabolomics, especially secondary product chemicals, which confer the functional properties of plants is dependent on its living environment [37]. Therefore, merely relying on DNA barcoding identification is insufficient for the quality control of herbal products. In addition, contamination in herbal products may not only be at the plant species level. DNA barcoding cannot authenticate low-quality herbal products, which arise when a non-prescribed plant part is used to substitute the prescribed part or when a prescribed plant part is not collected in the right season [2]. In the current research, the contents of TS-H and the functional compositions of *M*. *tenacissima* were determined by HPLC. The TS-H contents of the collected samples ranged from 0.39% to 1.09%, which is larger than that regulated in the Chinese Pharmacopoeia (0.12%) [21].

In summary, in the present work, the ITS2 barcode was coupled with TLC and HPLC to identify and evaluate the quality of *M*. *tenacissima* in herbal medicine markets. This study indicates that systems biology components encompass genomics (DNA barcoding) and metabolomics (for active secondary metabolites) for evaluating the quality of herbal medicinal materials and ensuring product safety in medicine markets.

## Supporting information

S1 Table Plant materials analyzed in this study. (PDF)
