# Supplementary material for: Integrated approach for identifying and evaluating the quality of Marsdenia tenacissima in the medicine market
Source: PLoS One. 2018 Apr 6;13(4):e0195240. doi: 10.1371/journal.pone.0195240 (PMC5889066; doi:10.1371/journal.pone.0195240)
Supplement: S1 Table — (PDF) [file pone.0195240.s001.pdf]

S1 Table. Plant materials analyzed in this study.

Species	Voucher NO.	GenBank NO.	Collection Place	Identification Result
M. tenacissima	TG001	KY964343	Drug store, Xi'an, Shanxi	Genuine
M. tenacissima	TG002	KY964344	Drug store, Xi'an, Shanxi	Genuine
M. tenacissima	TG003	KY964345	Drug store, Xi'an, Shanxi	Genuine
M. tenacissima	TG004	KY964346	Drug store, Xi'an, Shanxi	Genuine
M. tenacissima	TG005	KY964347	Drug store, Xi'an, Shanxi	Genuine
M. tenacissima	TG006	KY964348	Yuzhou Medicine Market, Yuzhou, Henan	Genuine
M. tenacissima	TG007	KY964401	Yuzhou Medicine Market, Yuzhou, Henan	Fake
M. tenacissima	TG008	KY964349	Yuzhou Medicine Market, Yuzhou, Henan	Genuine
M. tenacissima	TG009	KY964350	Yuzhou Medicine Market, Yuzhou, Henan	Genuine
M. tenacissima	TG010	KY964351	Drug store, Jiangxi	Genuine
M. tenacissima	TG011	KY964352	Anguo Medicine Market, Baoding, Hebei	Genuine
M. tenacissima	TG012	KY964353	Anguo Medicine Market, Baoding, Hebei	Genuine
M. tenacissima	TG013	KY964354	Anguo Medicine Market, Baoding, Hebei	Genuine
M. tenacissima	TG014	KY964355	Anguo Medicine Market, Baoding, Hebei	Genuine
M. tenacissima	TG015	KY964356	Anguo Medicine Market, Baoding, Hebei	Genuine
M. tenacissima	TG016	KY964357	Anguo Medicine Market, Baoding, Hebei	Genuine
M. tenacissima	TG017	KY964358	Anguo Medicine Market, Baoding, Hebei	Genuine
M. tenacissima	TG018	——	Anguo Medicine Market, Baoding, Hebei	Genuine
M. tenacissima	TG019	——	Drug store, Yunnan	Genuine
M. tenacissima	TG020	KY964359	Drug store, Yunnan	Genuine
M. tenacissima	TG021	KY964360	Drug store, Yunnan	Genuine
M. tenacissima	TG022	KY964361	Drug store, Yunnan	Genuine
M. tenacissima	TG023	KY964362	Drug store, Yunnan	Genuine

M. tenacissima	TG024	KY964363	Drug store, Yunnan	Genuine
M. tenacissima	TG025	KY964364	Drug store, Yunnan	Genuine
M. tenacissima	TG026	KY964365	Drug store, Yunnan	Genuine
M. tenacissima	TG027	KY964366	Drug store, Yunnan	Genuine
M. tenacissima	TG028	——	Bozhou Medicine Market, Bozhou, Anhui	Genuine
M. tenacissima	TG029	KY964367	Bozhou Medicine Market, Bozhou, Anhui	Genuine
M. tenacissima	TG030	KY964368	Bozhou Medicine Market, Bozhou, Anhui	Genuine
M. tenacissima	TG031	KY964369	Bozhou Medicine Market, Bozhou, Anhui	Genuine
M. tenacissima	TG032	KY964370	Bozhou Medicine Market, Bozhou, Anhui	Genuine
M. tenacissima	TG033	KY964371	Bozhou Medicine Market, Bozhou, Anhui	Genuine
M. tenacissima	TG034	KY964372	Bozhou Medicine Market, Bozhou, Anhui	Genuine
M. tenacissima	TG035	KY964373	Bozhou Medicine Market, Bozhou, Anhui	Genuine
M. tenacissima	TG036	KY964374	Drug store, Shanghai	Genuine
M. tenacissima	TG037	KY964375	Drug store, Shanghai	Genuine
M. tenacissima	TG038	KY964376	Drug store, Shanghai	Genuine
M. tenacissima	TG039	KY964377	Drug store, Shanghai	Genuine
M. tenacissima	TG040	KY964378	Drug store, Shanghai	Genuine
M. tenacissima	TG041	KY964379	Drug store, Guizhou	Genuine
M. tenacissima	TG042	KY964380	Drug store, Guizhou	Genuine
M. tenacissima	TG043	KY964381	Drug store, Guizhou	Genuine
M. tenacissima	TG044	KY964382	Drug store, Xingning, Guangdong	Genuine
M. tenacissima	TG045	KY964383	Drug store, Xingning, Guangdong	Genuine
M. tenacissima	TG046	KY964384	Drug store, Xingning, Guangdong	Genuine
M. tenacissima	TG047	KY964385	Drug store, Xingning, Guangdong	Genuine
M. tenacissima	TG048	KY964386	Drug store, Xingning, Guangdong	Genuine
M. tenacissima	TG049	KY964387	Drug store, Xingning, Guangdong	Genuine

M. tenacissima	TG050	KY964388	Drug store, Xingning, Guangdong	Genuine
M. tenacissima	TG051	KY964389	Drug store, Cheng du, Sichuan	Genuine
M. tenacissima	TG052	KY964390	Drug store, Cheng du, Sichuan	Genuine
M. tenacissima	TG053	KY964391	Drug store, Cheng du, Sichuan	Genuine
M. tenacissima	TG054	KY964392	Drug store, Cheng du, Sichuan	Genuine
M. tenacissima	TG055	KY964393	Drug store, Cheng du, Sichuan	Genuine
M. tenacissima	TG056	KY964394	Drug store, Yulin, Guangxi	Genuine
M. tenacissima	TG057	KY964395	Drug store, Yulin, Guangxi	Genuine
M. tenacissima	TG058	KY964396	Drug store, Yulin, Guangxi	Genuine
M. tenacissima	TG059	KY964397	Drug store, Yulin, Guangxi	Genuine
M. tenacissima	TG060	KY964398	Drug store, Yulin, Guangxi	Genuine
M. tenacissima	TG061	KY964399	Drug store, Yulin, Guangxi	Genuine
M. tenacissima	TG062	KY964400	Drug store, Yulin, Guangxi	Genuine
M. tenacissima	——	GQ434576.1	GenBank	——
T. cordata	——	AM980873.1	GenBank	——
F. polyanthum	——	KP096047.1	GenBank	——
T. sinensis	——	KR425497.1	GenBank	——
